# A Multiple Emergency Ventilator as Backup Solution in Pandemic: A Specifically Designed and Dimensioned Device

**DOI:** 10.1109/OJEMB.2022.3152673

**Published:** 2022-02-18

**Authors:** Giuseppe Baselli, Gianfranco B. Fiore, Francesco Casella, Simone Cinquemani, Roberto Viganò, Antonio Pesenti, Alberto Zanella

**Affiliations:** Politecnico di Milano 18981 20133 Milano Italy; Department of Electronics Information and BioengineeringPolitecnico di Milano 18981 20133 Milano Italy; Department of MechanicsPolitecnico di Milano 18981 20133 Milano Italy; Anestesia e Rianimazione DepartmentIRCCS Ca’ Granda Ospedale Maggiore Policlinico 9339 20122 Milano Italy

**Keywords:** COVID-19 pandemic, ICU mechanical ventilation, pressure-controlled and volume-guaranteed ventilation, assisted ventilation

## Abstract

*Goal:* To provide a Multiple Emergency Ventilator (MEV) as backup in case of shortage of ICU ventilators and for use in camp hospitals. *Methods:* MEV provides the same oxygen mixture and peak inspiratory pressure (PIP) to 10 patients. These specifications were fixed: i) gas supply and plugs to double-limb intubation sets compatible to existing systems; ii) fluid-dynamics with no pressure drop and almost complete patients’ uncoupling; iii) individual monitoring of inspiratory and expiratory pressures and flows and control of their timing; iv) easy stocking, transport, installation with self-supporting pipes. *Results:* A Bell-Jar System (BJS) design permitted to safely fix PIP based on Archimedes’ law. The main distribution line was based on 2” stainless steel pipes assuring the required mechanical properties and over-dimensioned for fluidics. The Windkessel of the BJS and pipeline dead-volumes is 75.65 L and in the worst case of the instantaneous demand of 5 L by 10 patients (0.5 L each) shows an adiabatic PIP drop limited to –6.18%, confirming the needed uncoupling. Consequently, patients’ asynchrony is permitted as needed by pressure-controlled volume-guaranteed and assisted-ventilation. *Conclusions:* Although MEV is proposed as a backup system, its features may cover the whole set of ventilation modes required by ICU ventilation.

## I. Introduction

As The COVID-19 pandemic was growing fast also the need for ICU ventilators stepped on. Unforeseen efforts were dedicated by research and industry to increase the number of ventilators and also to multiply the number of patients assisted by a single one by some adaptation at the patients’ end. However, medical societies and regulatory agencies in the US [1] and the EC [2], while easing the former direction strongly discouraged the latter one. Specifically, the US statement [1] fully included (Appendix B) a joint declaration of the main clinical societies in the field [3], which traced back the idea to 2006 [4] (see [3] for subsequent works) and clearly motivated their rejection on detailed technical and clinical reasons.

The Multiple Emergency Ventilator (MEV) proposed here was conceived on a totally different principle. Indeed, the mere adaptation of formerly existent equipment suffers from the intrinsic flow limitation of single patient ventilators and also is prone to uncontrolled flow partition to the two or more patients with different and unpredictable respiratory system properties. The cited regulations [1], [2] came next and did not address the new MEV concept; nonetheless MEV was attentively reconsidered to check all the specific issues there referred to adaptations not to jeopardize its rationale.

MEV strength resides in being purposely designed and dimensioned to safely assist 10 patients by positive pressure ventilation with oxygen mixture, concentrating and simplifying the major pressure control issues into a single common element, which was identified in a water sealed bell-jar system (BJS). In this way the peak inspiratory pressure (PIP) is intrinsically limited at (or slightly below) the BJS pressure, which in turn is intrinsically fixed by Archimedes’ law. High simplification of the single patient lines is permitted by the common pressure drive and by the distribution system allowing a full uncoupling among patients assisted in parallel. This feature was enhanced by the high over-dimensioning of the distribution pipe (2”), which also allows the wanted mechanical properties needed in an emergency environment: easy storing and assembling, self-supporting and support to all the related devices (e.g., monitors, local control sets, individual PEEP system).

It is due stating that MEV is proposed as a backup choice in emergency conditions, while single patient ventilators firmly remain the first option. Nonetheless, the recently passed COVID-19 peak has shown how the limits to cover the rising phase of crisis can be rapidly reached, even in western Countries. Also, new crises can be foreseen in this pandemic if vision is dutifully enlarged to developing Countries and to emergency conditions [5].

Indeed, the parallel option is to maintain huge reserves of ventilators with current design [6] and the ongoing projects of new simplified types [7]. Nonetheless, systems as the here presented Multiple Emergency Ventilator (MEV) may also play a role as backup options, easily recovered from stocks or applied in emergency times and/or places.

## II. Materials and Methods

In the first few days of work, the backbone of MEV was conceived with maximum simplification and functions limited to pressure-controlled ventilation, to be deployed to assist severely dyspneic patients waiting for a fully equipped ICU bed, potentially for several hours, given the rising load of these departments. Soon, we understood that the intrinsic volume buffering of MEV (see Results) permitted a decoupling of patients’ lines sufficient to permit higher level asynchronous functions such as assisted ventilation on each patient's demand. The enhanced version, here presented, is referred to as MEVplus in our internal open report [8] and widely broadened the potential applications at the expense of minimal additions in individual patient sensors and ventilation process control, as detailed in Supplementary Materials (SM) A.

### A. Design Specifications

The driving design specifications are listed below and referred next (S1, S2, …) in Results.

S1) Inflow O_2_ concentration (F_i_O_2_) necessarily equal for all patients, though adaptable to average needs.
S2) PIP necessarily equal to all patients, yet easily adaptable.
S3) Adoption of an intrinsically safe physical principle determining PIP against accidental barotrauma.
S4) Robust and simple design of the core element (BJS).
S5) Dimensioning of gas supply and distribution for up to 10 patients; all by O_2_-compatible elements.
S6) Negligible dynamic pressure drops in the common distribution line.
S7) Self-supporting gas distribution system (e.g., inside a camp hospital), giving an easy mounted support to all devices.
S8) Easy stocking, transport, installation, and maintenance.
S9) Flexible layout, fitting to any ambient shape.
S10) Individual expiration lines with adaptable PEEP device.
S11) Plug of standard double-limb intubation sets.
S12) Personalized ventilation timing, with a negligible pressure drop in the worst case of a simultaneous inspiration by all patients.
S13) Wide range of ventilation modes, from controlled to assisted ventilation.
S14) Adoption of off-the-shelf components, whenever possible.

In addition, prevention of cross infections by viral and bacterial agents is a core issue. MEV countermeasures are: i) each patient connected to a standard disposable ventilator circuit; ii) individual inspiration and expiration lines, the latter separated from the distribution system; iii) a nonreturn valve at both the inspiration and expiration ends; iv) easy dismantling of both lines for sanitization. No segmentation is conversely foreseen for the common 2” line; hence, the absence of back contamination via the inspiratory line should be attentively checked on the future prototype tests.

### B. Team and Project Management

Interdisciplinary cooperation coordinated by a Biomedical Engineer (G.B.) and to an Intensive Care MD (A.Z.). CAD of special parts was on two Mechanical Engineers (S.C. and A.V., see also SM-B). System fluid-dynamics and intrinsic safety feature were on a Bio-Mechanical Engineer (G.B.F.). System and control simulations were carried out by an Automation Engineer (F.C., see also SM-C). Focus onto the potential compliance with ICU clinical standards (to be tested and approved in the future development of MEV) was kept (A.P. and A.Z.).

### C. System Simulations

The model for system simulations was implemented in the Modelica language [9] and simulated with the open source OpenModelica tool [10]. A detailed description of the model and its control is provided in SM-C and [8].

The main body results present only the not recommended, most critical case of synchronous inspiration by 10 patients with non-restricted lung capacities to challenge decoupling capabilities (S12). Patients with different clinical conditions are considered, with airways resistance in the range *R* = 6÷18 cmH_2_0/(L/s) and lung compliance in the range *C* = 0.03÷0.06 L/cmH_2_0. The equivalent of the series of patient valves and pressure/flow measurement tools was simulated by a single valve flow coefficient *K_v_*=1.8 m^3^/h. The flow-dynamics of all distribution elements were modelled accounting for adiabatic compressibility (with coefficient γ = 1.4 for diatomic gas at room temperature) and friction effects, see Results. Tidal volumes ranging from 0.3 to 0.5 L were obtained, with RR ranging from 14 to 30 acts/min, depending on the patient conditions. Appropriate duty cycles were set to obtain the desired tidal volumes.

## III. Results

F_i_O_2_ as well as medical gas conditioning (S1) was not part of the project. Plug was assumed to standard medical gas lines (2-3 bar, relative) and gas conditioning.

### A. Pressure Stabilization By the Bell-Jar System (BJS)

**Fig. 1.**
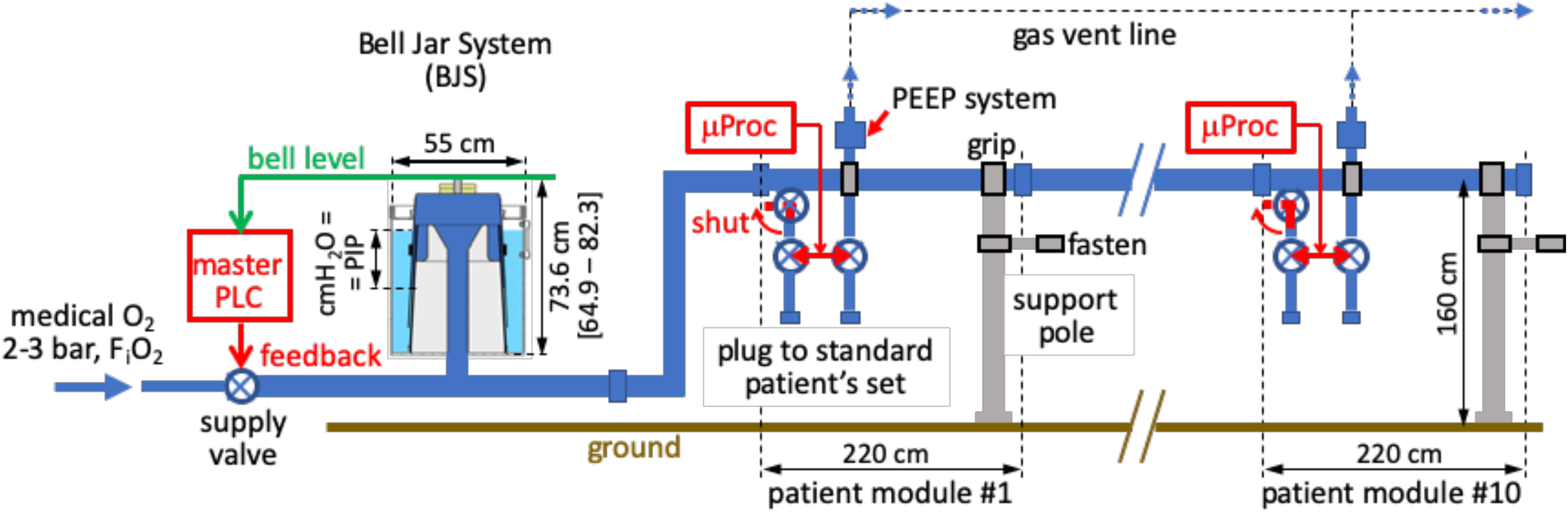
Scheme (out of scale) of the Bell-Jar System (BJS, larger scale), the O_2_ distribution system (blue), mechanical supports and grips (gray), BJS level measure (green), and controls (red). Legend of the BJS volumes: O_2_ (blue), air at atmospheric pressure (white), water-seal (light blue), and excluded volume (light gray). Note that the O_2_ volume is enlarged (dead volume) due to a water-trap ring (orange arrow) designed for safety.

**Fig. 2.**
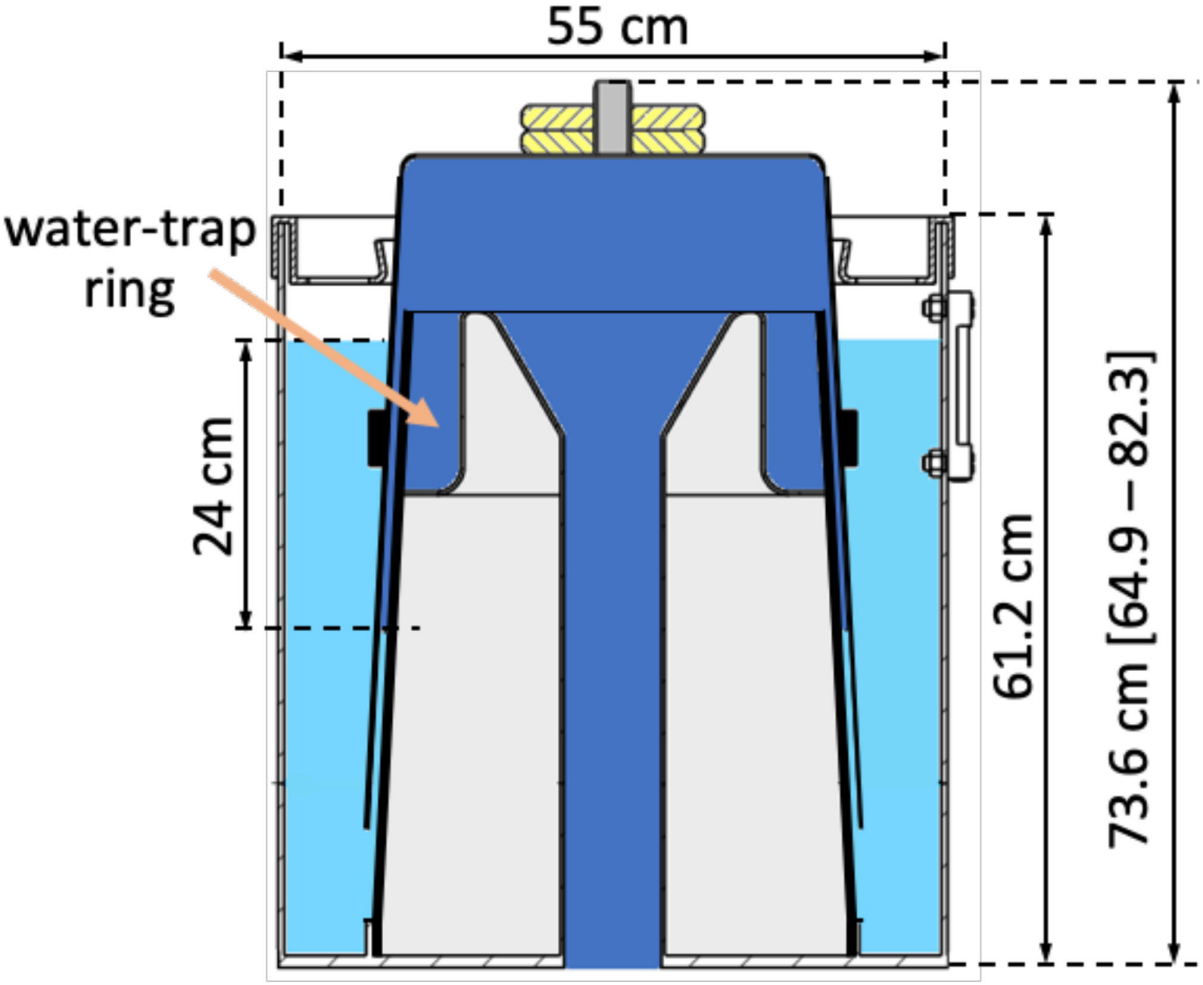
Bell-Jar System (BJS) CAD drawing at PIP = 24 cmH_2_O. Legend of BJS volumes: O_2_ (blue), air at atmospheric pressure (white), water-seal (light blue), and excluded volume (light gray). Note that the O_2_ volume is enlarged (dead volume) due to a water-trap ring (orange arrow) designed for safety.

Fig. 1 shows a scheme of the BJS, O_2_ mixture distribution, its mechanical support, and the main controls. The core supply element (S2-S5) is the water sealed BJS, detailed in Fig. 2). It fixes PIP upon gravity. The bell section at the set-point is 1166,7 cm^2^, which requires a bell-mass of 28 kg to provide the nominal PIP = 24 cmH_2_O. By subtracting the movable weights (yellow, in Fig. 2), the bell mass is reduced to 23.33 kg and PIP to 20 cmH_2_O. Mass augmented to 35 kg provides a PIP of 30 cmH_2_O. Stability and robustness against gripping was assured by the slightly conical shape of the bell and by its center of mass below the water level. Friction was limited by the water-seal itself, which avoids complex manufacturing. Stainless steel is foreseen for the bell and the internal elements and connector of the BJS. The external cylinder is foreseen in glass (or even Plexiglas, since protected from O_2_ by the water jacket) in order to assure visual check of the water seal levels and hence of PIP.

The dynamic range was fixed to ±10 liters (L), thus ±8.6 cm. Fig. 2 height dimensions consider the nominal condition PIP = 24 cmH_2_O (1 cmH_2_O = 0.98 millibar, relative), plus 1 cmH_2_O margin to the safety gas escape. Accordingly, a 24 cm level gap is shown between the internal water-seal level and the higher external one (see Fig. 6 of SM-B). Importantly, higher pressure values would result in a water-sealing loss, with the BJS O_2_ bubbling outside, thus assuring intrinsic safety and protection against barotrauma. Rising the operative range (and also the oxygen escape level) to 30 cmH_2_O implies increasing the BJS height dimensions by 6 cm.

The redundant BJS dead volume (59 L) was reduced by the central sealed element (44 L, light gray) to 15.27 L. The narrower cylinder at the sealed element top creates a water-trap preventing accidental water leakage to the core connector (blue).

### B. Main 2” Distribution Line

**Fig. 3.**
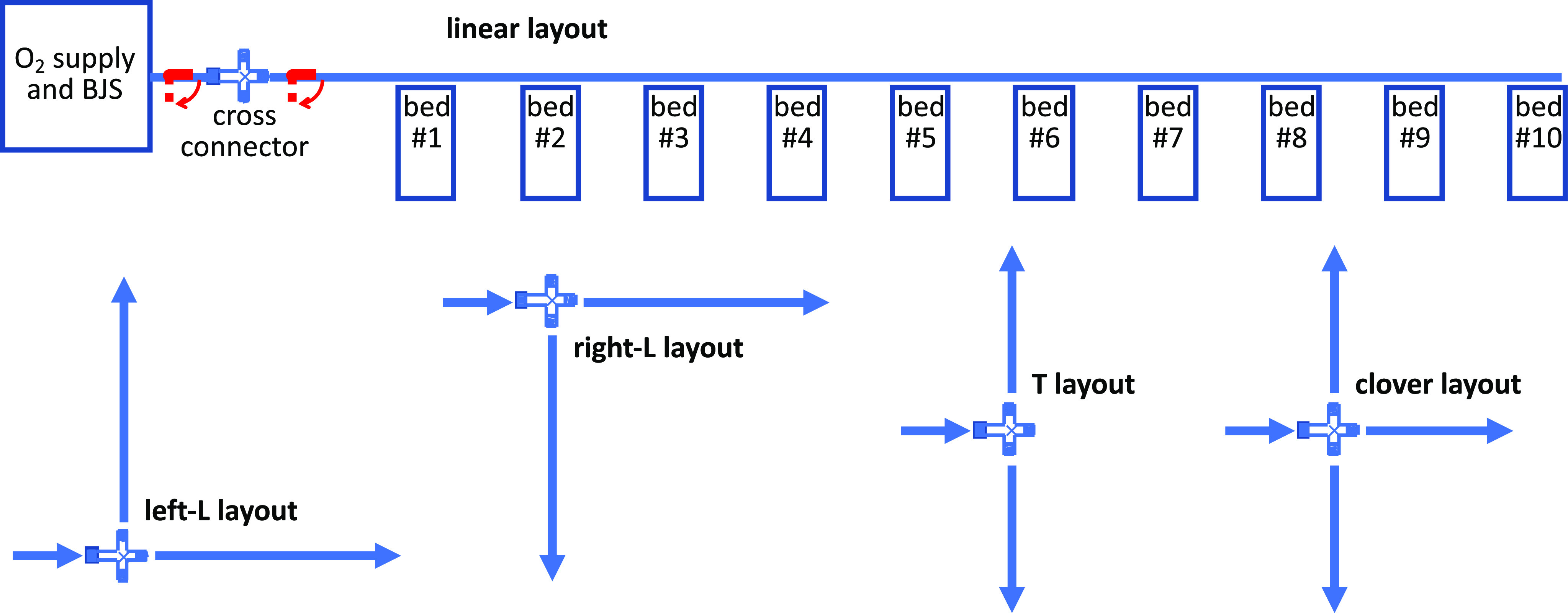
Schemes of the possible layouts.

Distribution (Fig. 1, S5-S9) was designed by modular elements of 2” stainless steel pipes. This apparently huge oversizing fully avoids gas transport pressure drops up to the last bed in a row of 10 (S6) and also provides the wanted mechanical self-supporting features (S7) by steel poles to be fixed to the wall or to beds. This design assures easy stocking, transport, assembly (S8), and flexible layouts (S9, see Fig. 3). It is suggested to let the 2” line run at 160 cm height, to be easily handled, though not interfering with the bed and related equipment. Each patient module is 220 cm wide and it is made by a 200 cm pipe and a 20 cm T-element with the 1/2” insertion of the inspiration line (see next Par.). The 1/2;” carries a manual shutter (red handles, in Fig. 1), to be maneuvered when attaching or detaching a patient.

The common gas supply to the BJS was dimensioned to 4 L/s maximum, which permits sufficient supply even in the case of the accidental detachment of a patient. For ergonomic reasons, the recommended installation is: i) female end of any element upstream; ii) the 20 cm T-element at the right bed-side, hence upstream to the 200 cm element. Additional connector and special elements are foreseen for the 2” distribution: short connectors with manual shutter to exclude the downstream distribution limb for maintenance; several sizes of linear elements (20, 50, 100, 200 cm); bows (90° and 45°); 20 cm element with water trap. Finally, a central cross element splits the inflow to three orthogonal lines which may be shut or active thus permitting the several layouts shown in Fig. 3 (S9): single linear, L or T shaped, clover-shaped. The dead volume in the 2” pipe is 2.03 L/m. An estimate of its total length is 2.2 m per patient times 10 = 22 m, plus about 3 m to the rows of beds.

### C. Inspiration and Expiration 1/2” Limbs

As said in the previous Par., the inspiration line is screwed (male to female) to a T-shaped element of the main line. It includes the series: i) inspiration/expiration switch valve, ii) flowmeter (not shown), iii) pressure meter (not shown), iv) one-way valve (not shown), and v) a standard 22 mm connector to the patient's intubation set.

Each independent expiration line (S10) is gripped (not connected) to the 2” pipe, at the right end of the 200 cm. It includes the series of: i) 22 mm plug to the intubation set, ii) one-way valve, iii) pressure meter, iv) flow-meter, v) switch valve, and vi) a standard adaptable PEEP element. It is recommended to collect the expired air by a vent line, properly dimensioned not to add overpressures to PEEP.

Plugging to standard double-limb patient sets (S11) imposed to have no element common to the inspiration and the expiration line; accordingly, inspiration/expiration change by a single three-way valve was forbidden. Thus, the two mentioned switch valves were inserted to be opened and closed in counterphase (see the two-end red arrow, in Fig. 1).

### D. Uncoupling of Patients and Consented Ventilation Modes

Results are here limited to the most critical case of simultaneous inspiration. A detailed description of modeling with more realistic examples is provided in SM-C.

Concerning the core condition of uncoupling among patients (S12), the worst case is the synchronous inspiration of 10 patients summarized below. Even more severe is an instantaneous adiabatic expansion. Though purely theoretical, it permits to set a lower bound to PIP drops, independent from patients resistive and capacitive features and the dynamic compensation by the BJS. We start from the system dead-volume of 75.92 L summing up: i) the 15.27 L of BJS dead volume; ii) the 10 L from BJS set-point to its minimum level; iii) 50.65 L of the 2” distribution system (25 m times 2.03 L/m). An adiabatic expansion (γ = 1.4) of 5 L (0.5 L per patient) to 80.92 L gave a pressure drop from 24 to 22.52 cmH_2_O; i.e., a deviation limited to –6.18%.

A more realistic simulation of the synchronous worst case, including all the fluid-dynamic elements, is here summarized (see SM-C for details). During inspiration, lung volume curves reach their 0.5 L target in less than 0.75 s, with the farthest patient receiving 8% less volume, which could be easily compensated by slightly increasing the duty cycle.

Transient pressure dips at the inspiratory line inlets are also limited. Patient #1 supply pressure reaches a minimum of 19.5 cmH_2_O after 30 ms from the valve opening, and gets back to 24 cmH_2_O within 100 ms. Patient #10 supply pressure drops to 18.5 cm H_2_O after 40 ms, recovering to 22.5 cmH_2_O after 100 ms, and then recovering the full PIP by the end of the inspiration act. The efficiency of the BJS pressure stabilization was demonstrated by a bell-level drop limited to –3.5 cm, safely away from the –8.6 cm lower bound.

The above noticeable results permitted to consider the advanced and adaptable ventilation modes (S13) needed during the whole course of invasive ventilation, starting with controlled and ending with assisted modes [11].

## IV. Discussion

### A. MEV Applicability in a Crisis Context

As depicted by the graphical abstracts, in a pandemic crisis the need of ICU beds may rapidly rise, as unfortunately demonstrated by COVID-19. Facing a lack of ventilators, MEV may, at least temporarily, cover this necessity. Its intrinsically safe low-pressure distribution system avoids compromises as to patients’ safety: *primum non nocere* (first, do no harm). The described design features also allow quick placement in almost any indoor or room or outdoor camp hospital mounting robust pieces, all passive, robust to transport or long stockage. Off-the shelf components [12] were addressed to ease the availability of spare parts or even to permit construction from scratch. The dual mechanical and gas transport function of the main 2” pipeline greatly simplifies the overall structure. Finally, the safe PIP controlled moved upstream in the single BJS allows to keep the single patient's inspiratory and expiratory limbs to their essentials.

After one year since the COVID-19 pandemic burst, its spread-out has been by far more dramatic than it could be envisaged starting this work. So far, focus has been on urgent issues such as infection spread prevention, collection of the currently available equipment, and, most importantly, the availability of trained intensive care personnel as well as therapies and vaccines. Conversely, the development of new ventilation technologies, even auxiliary ones as MEV, has been seemingly too slow to face the recent pandemic phases. Despite eminent and early issued indications of risk [1]–[3], several attempts went on for the adaptation of standard ventilators to assist more than one patient (alias, ventilator sharing, multiplex ventilation) [13], [14]. This has been a direct consequence of the shortage of such life saving devices posing the dramatic alternative of elevating the triage thresholds to ICU admittance and the debate on pros and cons of this solution is still open [15].

The proposed MEV aims at overcoming the limits of ventilator sharing: i) power and flow under-dimensioned on a single patient basis; ii) extensive fluid-dynamic coupling; iii) identical ventilation mode and timing; iv) shared ventilation monitoring and limitations in individual alarming; v) high cross contamination risks. Fixed PIP and FiO2 are, conversely, common limits of both the MEV and shared ventilator approaches, while PEEP can be individually adapted in both systems. However, a novel device as MEV will have to undergo all the necessary development phases: engineering, technological transfer, validation, and approval for medical use. So, authors are stressing that the presented results are just a promising starting point. The major result of patients’ uncoupling is also easing the foreseen monitoring and control features as summarized in SM-A.

MEV was dimensioned for 10 patients mainly to have a group suited to the surveillance by a single medical staff member (nurse or MD). However, MEV dimensioning could be easily adapted, given its mechanical and monitoring modularity. Layout flexibility permits the best positioning of bed rows watching the individual monitors. The pressure and flow sensors on both the inspiratory and expiratory lines permit all the alarming options of common ventilators, the main of which would alert about tidal volume out of the set range.

### B. MEV Fluid-Dynamic Features

The oversizing of the main pipe section and dead-volume were imposed by the mechanical and ease-of-handling features. Nonetheless, they also permitted the favorable fluid-dynamic response shown in the results for the worst case of the farthest patient (#10 in a linear arrangement, 25 m away from the BJS) when inspiration was started in synchrony with the 9 ones, upstream and with demanding tidal volume and respiratory rate (RR) settings. The theoretical lower bound of PIP drop (instantaneous adiabatic expansion) limited to –6.18% provided a general result, independent from cycle timing and pulmonary patients’ features. This permits to conclude that the asynchronous modes discussed in the next Par. and SM-A are feasible under general operative conditions.

### C. Proposed Ventilation Modes

The main functional goal was to reach independently personalized controlled and assisted mechanical ventilation. This includes, single patient adaptation of the individually set tidal volume, PEEP, inspiration/expiration duty cycle, and single patient adaptation of RR.

As to pressure-controlled, volume-guaranteed mode, the physician will be allowed to input on the bed touchscreen the aimed tidal volume. The system will slowly (time to set-point = 1 min, approximately) adapt the inspiration/expiration duty cycle from the nominal value (1:2, expiration/inspiration) within the admitted range (1:1–1:3), keeping alarm until the set tidal-volume is reached.

Assisted-ventilation will be triggered by patient determined pressure/flow values. Briefly, i) a pressure drop in the inflow sensor will trigger the inspiration valve opening; ii) inflow equals to a predetermined percentage of the maximum peak of inflow or the reaching of a predetermined maximum inspiration time will trigger the switch to expiration. Alarm to be given if any of the above phase timed out or if the preset tidal volume and/or flow/min was not reached.

The main feature of ventilation strategies imposed by a fixed PIP permits the sole adaptation of time settings, mainly the duty-cycle. This obviously limits the applicable pressure-volume cycles, compared to those settable in a full ventilator. However, this limit is in favor to maximum safety against barotrauma.

Limits due to FiO_2_ and PIP common to all patients are foreseen to significantly constrain MEV application protocols. High FiO2 levels (e.g., 90-100%) can be considered in the first days of severe pneumonia treatment, starting with pressure-controlled, volume guaranteed mode and ending with assisted ventilation. However, the availability of ventilators would suggest moving recovering patients to standard treatment, as soon as possible. The same would be necessary for very severe patients to whom a fixed PIP could be not applicable. In case of extreme shortage, the only solution could be moving the recovering patients to a MEV delivering lower FiO_2_ levels (e.g., 50%). Technical solutions with personalized O_2_ mixtures could be easily conceived; however, the crisis contexts for which MEV is proposed suggest to attentively consider the tradeoffs between higher flexibility and instrumentation essentiality. In summary, the MEV backup is hopefully addressed to partial substitution of missing ventilators.

## V. Conclusion

The proposed MEV system was conceived at the highest peak of pandemic increasing rate in Northern Italy to overcome limits of adaptations of ICU ventilators to two or more patients and to provide a backup option to the lack of full ventilators, which are the first option. In this short presentation, the feasibility of the proposed system was shown, highlighting points of strengths relevant to robustness, intrinsic safety, and flexibility of each single patient ventilation mode. Intrinsic limits relevant to a common inspiration pressure and oxygen concentration can be foreseen as sufficiently compensated by the high gain in the equipment simplicity and sensibly lower costs.
